# DNA methylome responses to biotic and abiotic stress in *Arabidopsis thaliana*: A multi‐study analysis

**DOI:** 10.1111/plb.70291

**Published:** 2026-09-14

**Authors:** R. Behl, J. J. Gallo‐Franco, R. R. Hazarika, Z. Zhang, F. Wilming, J. P. Schnitzler, C. Lindermayr, F. Johannes

**Affiliations:** ^1^ Research Unit Environmental Simulation (EUS) Helmholtz Munich Neuherberg Germany; ^2^ International Center for Tropical Agriculture (CIAT), CGIAR Cali Colombia; ^3^ Plant Epigenomics, TUM School of Life Sciences Technical University of Munich Freising Germany; ^4^ Ecosystem Physiology, Faculty of Environment and Natural Resources University Freiburg Freiburg Germany; ^5^ Institute of Lung Health and Immunity (LHI), Comprehensive Pneumology Center (CPC‐M), Helmholtz Munich Neuherberg Germany; ^6^ TUM School of Life Sciences Technical University Munich Weihenstephan Germany

**Keywords:** *Arabidopsis thaliana*, DNA methylation, environmental stresses, epigenetics, inheritance, transposable elements

## Abstract

- Rationale: Plants experience diverse biotic and abiotic stresses that can induce changes in DNA methylation. However, comparisons among existing studies are complicated by differences in analytical methods and experimental designs. We aimed to identify shared and stress‐specific DNA methylation responses across studies.
- Methods: We reanalysed 16 whole‐genome bisulphite sequencing datasets from 13 Arabidopsis thaliana studies using a unified bioinformatics pipeline. Differentially methylated regions (DMRs) were assessed in the CG, CHG and CHH contexts and examined in relation to genes, transposable elements (TEs), Gene Ontology terms, gene‐proximal TE superfamilies and epimutation‐prone loci.
- Results: Global methylation levels were generally stable, whereas the number and genomic distribution of DMRs varied according to stress type and methylation context. CG‐DMRs occurred primarily in gene bodies, while CHG‐ and CHH‐DMRs were enriched in TEs. Functional analysis identified shared stress‐related processes across conditions. Gene‐proximal LINE/L1, RathE1_cons and DNA/HAT elements were enriched for stress‐responsive methylation changes. A subset of stress‐associated CG‐DMRs overlapped loci known to accumulate stable epimutations over generations.
- Conclusions: Standardized cross‐study analysis revealed both shared and stress‐specific methylation patterns. The association of stress‐responsive DMRs with gene‐proximal TEs and epimutation‐prone loci suggests potential links among environmental responses, genome regulation and long‐term epigenetic variation.

## Graphical abstract

Sixteen *Arabidopsis thaliana* Whole‐Genome Bisulfite Sequencing datasets have been analysed to reveal common and stress‐specific DNA methylation responses. Although global methylation levels remained stable, specific DMRs varied according to the type of stress and genomics context.

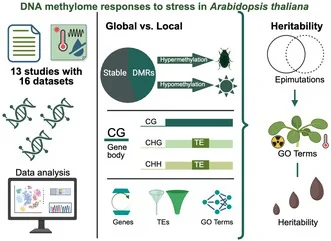

## INTRODUCTION

Climatic changes are increasing the frequency and intensity of environmental stresses (Schillerberg & Tian 2024; Gebrechorkos *et al*. 2025). As sessile organisms, plants must rapidly sense and adapt to a wide range of abiotic and biotic stressors, including drought, salinity and extreme temperatures, as well as pathogen attack (Jiang *et al*. 2025). A key mechanism underlying these adaptive responses is DNA methylation, an epigenetic modification that regulates gene expression and TE activity (Law & Jacobsen 2010). In plants, methylation occurs in CG, CHG and CHH sequence contexts (where H = A, T, C) and is catalysed by distinct sets of DNA methyltransferases (*e.g*., MET1, CMT2/3, DRM2), while active demethylation is carried out by enzymes such as ROS1 and DML2/3 (Finnegan *et al*. 2000; Finnegan & Kovac 2000; Dalakouras *et al*. 2012; Zhang *et al*. 2018; Sun *et al*. 2022).

DNA methylation plays diverse roles depending on its genomic context. In gene promoters, it typically represses transcription by blocking transcription factor access, while gene body methylation (gbM)—usually in the CG context—is associated with moderately expressed housekeeping genes and may stabilize transcription (Muyle *et al*. 2022). Methylation within TEs is especially important for silencing their activity and maintaining genome integrity. Emerging evidence also suggests that gene‐proximal TEs have been co‐opted during evolution and domestication as environmentally responsive regulatory elements (Dubin *et al*. 2018; Roquis *et al*. 2021; Ito 2022). These elements may act as tunable modulators of gene expression, with stress‐induced differential methylation serving as a mechanism for fine‐tuning transcriptional responses to environmental cues (Dubin *et al*. 2015).

A growing body of evidence supports a regulatory role of DNA methylation in stress acclimation (Bräutigam & Cronk 2018; Zhang *et al*. 2018; Kumar & Mohapatra 2021). For instance, in *Arabidopsis thaliana*, salt stress leads to demethylation of the AtMYB74 promoter, resulting in its transcriptional activation—a response likely essential for stress tolerance (Xu *et al*. 2015; Bartels *et al*. 2018). In the context of biotic stress, pathogen exposure can trigger dynamic shifts in CHH methylation and alter the expression of key defence‐related genes. This has been observed in Arabidopsis infected with *Pseudomonas syringae*, which exhibits both genome‐wide hypomethylation and local methylation changes at stress‐responsive loci (Pavet *et al*. 2006). Work with mutants defective in key DNA methylation enzymes, such as MET1, DRM2 and CMT3, further shows altered resistance to pathogens, indicating that proper methylation homeostasis is critical for immune responses (Slaughter *et al*. 2012; Zhang 2012).

DNA methylation changes induced by stress are not always transient. In some cases, they can be maintained across generations, contributing to transgenerational epigenetic inheritance (Johannes & Schmitz 2019). For example, under salt stress, hypomethylation of the transposable element *AT5TE35120* near the *CNI1* locus is stably inherited through the male germline and associated with the activation of the long non‐coding RNA *CNI1‐AS1*. Such cases suggest that stress‐responsive methylation changes may play a dual role—enabling immediate gene regulatory flexibility while also contributing to longer‐term heritable adaptations (Wibowo *et al*. 2016, 2018).

While many studies have explored DNA methylation changes in plants under stress using Whole‐Genome Bisulfite Sequencing (WGBS), their findings are difficult to compare directly (Table 1). This difficulty arises not only from methodological differences, such as divergent analysis pipelines, thresholds for identifying differentially methylated regions (DMRs) and different units of analysis, but also from differences in experimental design. These include stress type, stress intensity, exposure duration, recovery period, sampled tissue, developmental stage, plant genotype or accession and replicate structure. Such biological and technical heterogeneity can strongly influence the number, direction, genomic distribution and functional interpretation of stress‐associated methylation changes. Therefore, a standardized reanalysis of available datasets provides a useful framework for identifying methylation features that are robust across studies, while recognizing that some responses may remain stress‐, tissue‐ or stage‐specific.

**Table 1:** Overview of datasets analysed for DMR identification under various stress conditions in *Arabidopsis thaliana*.

study	stress type	stressor	tissue	developmental age	stress duration	code	reference	replicates	SRA Nr.	RNA seq
1	Abiotic	Gamma radiation	Leaf	Irradiation (Y110 = 110 mGy/h) started at 7 day and harvested at 21 days old	14 days	GR	(Laanen *et al*. 2021)	5	PRJNA665466	No
2	Abiotic	Senescence	Leaf	Rosette leaf 5 and 6 were covered with black cloth mitten on 42nd day and leaves were harvested on day 48	6 days	SE	(Trejo‐Arellano *et al*. 2020)	2	PRJNA545711	Yes
3	Biotic	Tobacco rattle virus	Leaf	Plants were rub‐inoculated on rosette leaves using fresh inoculum prepared from *Nicotiana benthamiana* leaves systemically infected with TRV‐GFP at 20 days post germination	14 days (for bisulphite sequencing)	TRV	(Diezma‐Navas *et al*. 2019)	1	PRJNA482577	No
4a	Abiotic	Phosphate starvation—Root tissue	Root	Seeds were germinated and grown either hydroponically for 7 days and then transferred to low phosphate (5 μM) KH_2_PO_4_ in liquid for 17 days. At day 17, root tissues were harvested	10 days	PST	(Yong‐Villalobos *et al*. 2015)	1	PRJNA295025	Yes
4b	Abiotic	Phosphate starvation—Shoot tissue	Leaf	Seeds were germinated and grown hydroponically for 7 days in high phosphate and then transferred to low phosphate (5 μM) KH_2_PO_4_ in liquid for 17 days. At Day 17, rosettes were harvested	10 days	PST	(Yong‐Villalobos *et al*. 2015)	1	PRJNA295025	Yes
5a	Abiotic	Heat	Leaf	At 21 day of growth, seedlings were subjected to heat stress (42 °C) for 24 h and then transferred back to 21°C for 24 h recovery. Leaf tissues were collected from plants after heat stress and after recovery	24 h	HE1	(Korotko *et al*. 2021)	3	PRJNA587704	No
5b	Abiotic	Heat recovery	Leaf	At 21 day of growth, seedlings were subjected to heat stress (42°C) for 24 h and then transferred back to 21°C for 24 h recovery. Leaf tissues were collected from plants after heat stress and after recovery	24 h	HE1‐Rec	(Korotko *et al*. 2021)	3	PRJNA587704	No
6	*Biotic*	*Pseudinomas syringae*	Leaf	Bacterial growth assays were performed in plants infected at 1 × 105 cfu.mL − 1 (OD600, 0.0002‐ for final infection) by vacuum infiltration. Leaves were harvested at 5 days post infection	5 days	PSY	(Dowen *et al*. 2012)	2	SRP012576	Yes
7	Abiotic	Hyperosmotic	Leaf	Seeds from a single founder plant were germinated and grown on MS medium (control) for 2 weeks and transferred to MS medium supplemented with 75 mM NaCl to induce mild hyperosmotic stress for 4 weeks. Before flower buds were visible, plants were transferred to soil. Leaf and seed samples were collected from 10‐week‐old plants. We used G5 P0 for our analysis	10 weeks	OS	(Wibowo *et al*. 2016, 2018)	2	PRJEB9076	No
8	Abiotic	Heat	Leaf	Control plants were grown in 1993 temperature profile from Munich, Germany and heat‐treated plants in 2003 temperature profile from Munich, Germany. Leaf samples were harvested at 4.5 weeks post germination	4.5 weeks	HE2	Unpublished	2	LRZ	No
9	Abiotic	Heat	Leaf	Heat stress was applied at 45°C for 4 h for 4‐days starting at day 10 post germination. Progeny of 25th generation were grown for 21 days and rosette tissues from these were harvested for bisulphite sequencing	4 h/day for 4 days	HE3	(Yadav *et al*. 2022)	5	PRJNA802915	No
10	Abiotic	UV exposure	Leaf	After 21 days of post germination, plants (40 plants/plate) were irradiated with UV‐C (3,000 J/m^2^) using Stratalinker. Leaf samples were collected at time 0 (immediately after UV exposure) and after 24 h of UV exposure	24 h	UV	(Graindorge *et al*. 2019)	1	PRJNA548919	No
11a	Abiotic	Cold stress—High light	Leaf	Plants were grown in a controlled growth chamber set to 10°C, 500 μE m − 2 s − 1 and 12/12 day/night photoperiod for 21 days, beginning from germination, then moved to recovery at 22°C, 250 μE m − 2 s − 1 for 18 days before sampling	21 days	CO‐CHL	(Kenchanmane Raju *et al*. 2018)	2	PRJNA482906	Yes
11b	Abiotic	Cold stress—Low light	Leaf	Plants were grown in a controlled growth chamber set to 10°C, 150 μE m − 2 s − 1 and 12/12 day/night photoperiod for 21 days, beginning from germination, then moved to recovery at 22°C, 250 μE m − 2 s − 1 for 18 days before sampling	21 days	CO‐LL	(Kenchanmane Raju *et al*. 2018)	2	PRJNA482906	Yes
12	Abiotic	Drought	Leaf	The first treatment was applied at 1 week of age, which involved saturating soil moisture and subsequently withholding water for 2 weeks. Plants were then watered and allowed to recover for 5 days. The second treatment was repeated following recovery, although this time for only 12 d to minimize plant death. We used data from generation 5 which was exposed to repeated exposure to drought and leaf samples from Week 4 old plants	2 weeks +12 days	DR	(Ganguly *et al*. 2017)	5 (control), 6 (stress)	PRJNA368978	
13	Abiotic	Salinity	Leaf	The groups were planted in soil, three groups in soil saturated with 125 mM NaCl (Saline soil) and three groups in control, water‐saturated soil (Control). We used data from generation 10 for analysis	Throughout for 10 generations	SA	(Jiang *et al*. 2014)	3	SRP047267	

To overcome this challenge, we conducted an integrative reanalysis of 16 Whole‐Genome Bisulfite Sequencing (WGBS) datasets from 13 published *Arabidopsis thaliana* studies using a standardized bioinformatic pipeline to identify common and stress‐specific methylation responses. By harmonizing DMR detection and annotation across studies, we aim to identify both common and condition‐specific methylation responses, with particular focus on stress‐regulated genes and TEs. This unified framework provides a more consistent view of the epigenetic landscape underlying stress responses and lays the groundwork for identifying conserved regulatory features and potential targets to improve stress resilience in crops.

## MATERIALS AND METHODS

### Data collection: Inclusion and exclusion criteria

WGBS datasets related to *A. thaliana* stress responses were searched and downloaded from the NCBI‐SRA database (https://www.ncbi.nlm.nih.gov/sra). Following the SRA search, datasets were screened by reviewing the experimental design and associated publications. Studies were included if they met the following criteria: (i) WGBS data were available for *A. thaliana*, preferably ecotype Col‐0; (ii) the study contained a clearly defined comparison between control and stressed plants; (iii) the stress condition represented an abiotic or biotic environmental perturbation; and (iv) sufficient metadata were available to identify the control and treatment samples. Datasets focusing on other types of epigenetic marks, such as N^6^‐methyladenosine (m^6^A), were excluded.

Because publicly available *Arabidopsis* stress methylome datasets remain limited, we did not restrict the primary analysis to studies with identical stress duration, stress intensity, tissue type or developmental stage. Applying such strict criteria would have substantially reduced the number of eligible datasets and limited the ability to identify broader methylation patterns across stress conditions. Instead, our main objective was to minimize computational heterogeneity by reprocessing all available datasets with a unified bioinformatic pipeline. At the same time, experimental heterogeneity was documented and accounted for in the interpretation. Metadata describing stress type, stress intensity, exposure duration, recovery period, tissue, developmental stage, genotype/accession, replicate number and sequencing design are provided in Table 1. In total, 16 datasets from 13 studies met the inclusion criteria and were compiled for further analysis (Fig. S1).

### WGBS data processing

WGBS data was processed using the MethylStar pipeline, which integrates tools for read trimming (Trimmomatic), alignment (Bismark) and cytosine‐level methylation calling (Methimpute) as described in (Taudt *et al*. 2018; Shahryary *et al*. 2020) and https://github.com/jlab‐code/MethylStar.

### Methylation level analysis

Global methylation levels were calculated as the average genome methylation across the genome. This quantity reflects the approximate proportion of cytosines that are methylated at the genome‐wide scale. Statistical comparisons between control and stress conditions were performed on the global methylation data using an unpaired *t*‐test where more than one replicate was available. For datasets with only a single replicate, the Wilcoxon rank‐sum test (Mann–Whitney *U* test) was applied. Multiple testing adjustments were conducted using the Bonferroni and Holm‐Bonferroni methods, with a significance threshold of *P* < 0.05.

### DMR analysis

DMRs were identified using jDMR (Hazarika *et al*. 2022) (https://github.com/jlab‐code/jDMR). To analyse cytosine clusters, the genome was divided into 500‐bp sliding windows for CG and CHG contexts and 100‐bp windows for CHH contexts. Only bins with at least ten cytosines were retained. Methylation region calls were obtained for all three contexts (CG, CHG and CHH) using sliding windows. Hence, the methylation status of a given region was classified as either unmethylated or methylated. Pairwise comparisons between the control and stress conditions were conducted by generating a DMR matrix containing region calls corresponding to each sample and each bin. Non‐epipolymorphic patterns were excluded, and adjacent bins were merged to obtain the final list of DMRs. The number of DMRs within each window was calculated for each study and sequence context, and DMR density was plotted to visualize the distribution of methylation changes across the genome.

### DMR‐associated genes and transposable elements (TEs) analysis

Annotation files for TEs, genes and promoters were obtained from TAIR 10, and the intergenic region was delineated using Bedtools. DMR‐associated TEs, promoters, intergenic regions and genes were identified for each study, with more than 60% overlap between a DMR and the TE, gene body or promoter. Shared DMR‐associated genes and TEs among stress conditions were calculated and visualised to highlight similarities or differences across stress conditions. Chi‐square tests were then used to assess whether annotation distributions differed across methylation contexts and within each context across studies. Pairwise study comparisons were performed within each methylation context and adjusted using the Benjamini–Hochberg method, and Cramer's V was calculated as an effect size.

Gene Ontology (GO) enrichment analysis was performed on DMR‐associated genes using g:Profiler (FDR = 0.05) (Raudvere *et al*. 2019) and https://biit.cs.ut.ee/gprofiler/gost. Thereafter, shared enriched GO terms were compared using the GO Compare graph algorithm for CG, CHG and CHH contexts (Sosa *et al*. 2023). The distance of each TE to its nearest gene was calculated. These distances were then categorized into four quartiles based on gene‐TEs proximity (25% quartile—1119.25 bp or ~1.2 kb, 50% quartile 1721 bp or ~1.7 kb, 75% quartile—2568.75 bp or ~2.57 kb and 100% quartile—9998 bp or ~10 kb). TEs were further classified into superfamilies, and enrichment analysis of TE families within each quartile was conducted. The closest 25% quartile of TEs, with the proximity distance of ~1.2 kb to genes, was selected for further analysis. TE superfamilies within this 25% quartile were extracted, and their enrichment was calculated as the total base pair overlap with DMRs relative to the total TE space. Resulting values were normalized using min–max scaling. Genes located within ~1.2 kb of these TEs were subjected to GO analysis using g:profiler (FDR = 0.05) to identify potential pathways influenced by TE‐associated methylation. All calculations and visualizations were carried out in R and RStudio, with plot adjustments performed in Inkscape.

### Metadata‐stratified subgroup analysis

To address experimental heterogeneity among the included WGBS datasets, we performed an additional metadata‐stratified analysis using subsets of studies with more comparable stress or tissue characteristics. Based on the curated experimental metadata in Table 1, datasets were grouped into four subgroups: heat‐stress, phosphate‐starvation, biotic‐stress and leaf‐derived samples. The heat subgroup included HE‐1, HE‐1 Recovery, HE‐2 and HE‐3; the phosphate subgroup included PST‐R and PST‐S; the biotic subgroup included TRV and PSY; and the leaf subgroup included datasets derived from aerial or leaf tissue.

For each subgroup, DMR‐associated genes and TEs were compared across studies separately for CG, CHG and CHH contexts. Functional recurrence within subgroups was assessed using g:Profiler enrichment outputs. To further evaluate whether recurrent methylation changes affected potentially regulatory TE annotations, we focused on TEs located near genes. Fisher's exact tests were used to test whether subgroup‐consensus gene‐proximal DMR‐associated TEs were enriched for specific TE superfamilies. *P*‐values were adjusted using the Benjamini–Hochberg method.

### Analysis of spontaneous epimutation rates

To evaluate whether a subset of stress‐responsive loci are capable of accumulating transgenerationally stable methylation changes, we analysed spontaneous CG epimutation rates in wild‐type *Arabidopsis thaliana* (Col‐0) mutation accumulation (MA) lines using the AlphaBeta R package (v1.10.0) ((Shahryary *et al*. 2020) and https://github.com/jlab‐code/AlphaBeta). We first compiled CG‐associated DMRs from multiple stress studies into a unified annotation set. These regions were then assessed in the MA_Col‐0 dataset, which includes WGBS data from two independent lineages spanning 17 generations ((Yao *et al*. 2023); GEO accession GSE223810). Following the approach of Hazarika *et al*. (2022), we estimated the CG methylation gain (α) and loss (β) rates with AlphaBeta. This method builds on earlier modelling frameworks (*e.g*., Van Der Graaf *et al*. 2015) and uses multi‐generational WGBS data along with known pedigree structures to estimate epimutation rates and their confidence intervals.

## RESULTS

To identify stress‐specific and conserved changes in DNA methylation, we performed a comparative analysis of studies (reported in Table 1) to further reveal the function of DNA methylation in plant stress response using a common bioinformatic approach.

### Global DNA methylation levels are stable under stress

Published *A. thaliana* DNA methylation studies using biotic or abiotic stress were extracted from the NCBI‐SRA database. In total, 13 publications were identified (Fig. 1A) that meet our search criteria (see Methods). These studies included abiotic stressors such as senescence (SE), gamma radiation (GR), phosphate starvation (PST)‐root (R) and shoot (S), high osmolarity (OS), UV radiation (UV), cold low light (CO‐LL) and high light (CO‐CHL), drought (DR), Salinity (SA) and enhanced temperatures (3 studies, HE‐1, HE‐2 and HE‐3 with HE‐1Rec, which represents plants that recovered from heat stress) as well as biotic stresses such as Tobacco Rattle Virus (TRV) and *Pseudomonas syringae* (PSY). We re‐analysed the DNA methylation data of these studies using a uniform analysis pipeline (see Methods). A comparison of global CG, CHG and CHH methylation levels between treatment and control groups did not reveal statistically significant differences in any of the studies, except for DR (*P* = 0.003) in the CHG context (Fig. 1B and Table 2). These results show that global steady‐state DNA methylation is robustly maintained in response to biotic or abiotic stress. However, a global analysis potentially masks stress‐induced hypo‐ or hypermethylated DMRs at specific genomic regions. Their effects may not be detectable in global averages, either because there are too few in numbers, or because hyper‐ and hypomethylated DMRs occur with similar frequencies. To assess this, we mapped DMRs in each study.

**Fig. 1:**
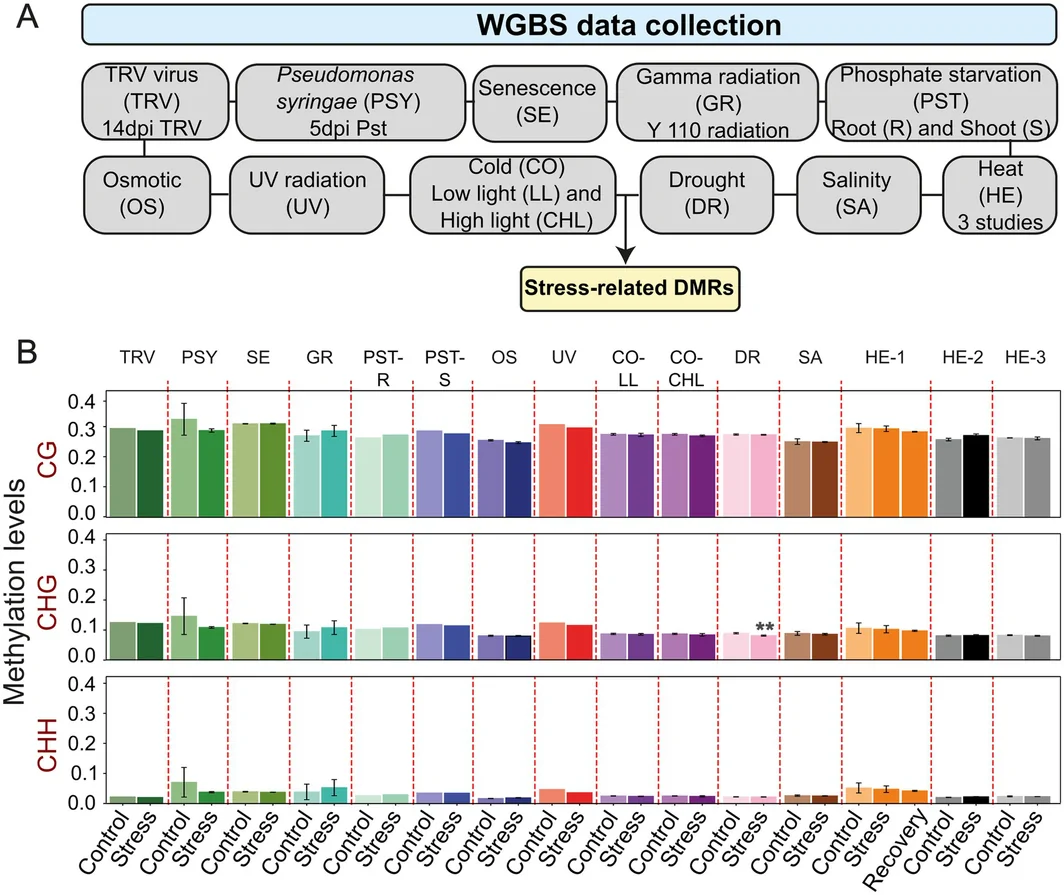
Overview of analysed studies and methylation levels in different sequence contexts. (A) Graphical representation of the experimental conditions analysed for various studies, including biotic stresses (*e.g*., *Pseudomonas syringae* infection and TRV virus), abiotic stresses (*e.g*., UV radiation, drought, salinity, heat and phosphate starvation) and developmental cues (*e.g*., senescence). (B) Average methylation levels for stress‐related methylomes across CG, CHG and CHH sequence contexts. Each bar represents the mean methylation percentage for the respective context in a specific study, with red dashed lines separating individual experimental conditions. Error bars indicate standard deviations.

**Table 2:** Statistical analysis of global methylation differences across studies for all three contexts—CG, CHG and CHH.

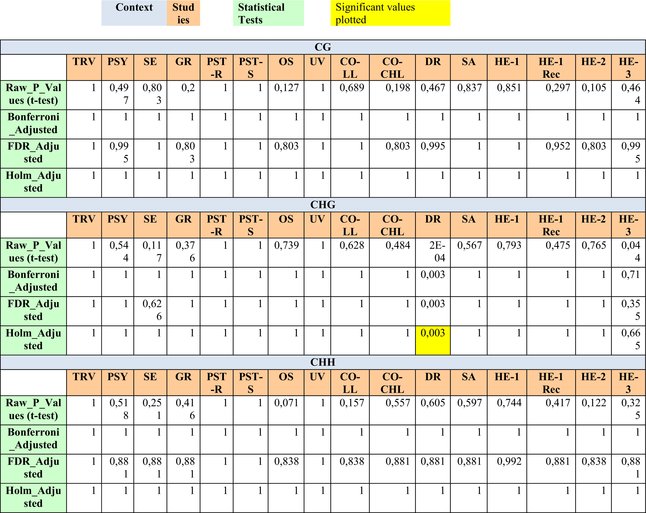																

The table presents the results of *t*‐tests comparing control and stress conditions for each study, followed by multiple testing adjustments (Bonferroni, FDR and Holm methods). Each row represents a statistical test, and columns correspond to studies. Significant values plotted (highlighted in yellow) indicate adjusted *P*‐values (<0.05) below the significance threshold. Contexts (CG, CHG and CHH) are labelled distinctly to showcase the corresponding methylation analysis.

### Differentially methylated regions (DMRs) associated with stress conditions

Hypermethylated DMRs were defined as genomic regions that showed local increases in methylation under stress relative to controls. Similarly, hypomethylated DMRs were defined as regions displaying local methylation losses (Fig. 2A; Fig. S2A–C). We identified considerable variation in the total number of DMRs across the studies, ranging from 52 to ~13.5 k (Fig. 2A). To statistically evaluate whether the direction of methylation change differed among studies, we calculated the proportion of hypermethylated DMRs for each study and methylation context and tested deviations from a 50:50 hyper: hypomethylation expectation using binomial tests with Benjamini–Hochberg correction. In addition, pairwise *post hoc* proportion tests were performed within each methylation context to compare DMR directionality across studies. This analysis showed that the balance between hyper‐ and hypomethylated DMRs was highly context‐ and study‐dependent (Fig. 2B). In the CG context, several datasets showed significant enrichment of hypermethylated DMRs, including CO‐CHL, HE‐1, HE‐1 Recovery, PST‐S, PSY, TRV and UV, whereas GR, DR, HE‐2 and PST‐R showed a bias towards hypomethylation. CO‐LL, OS and SA did not show a significant deviation from a balanced hyper–hypo ratio.

**Fig. 2:**
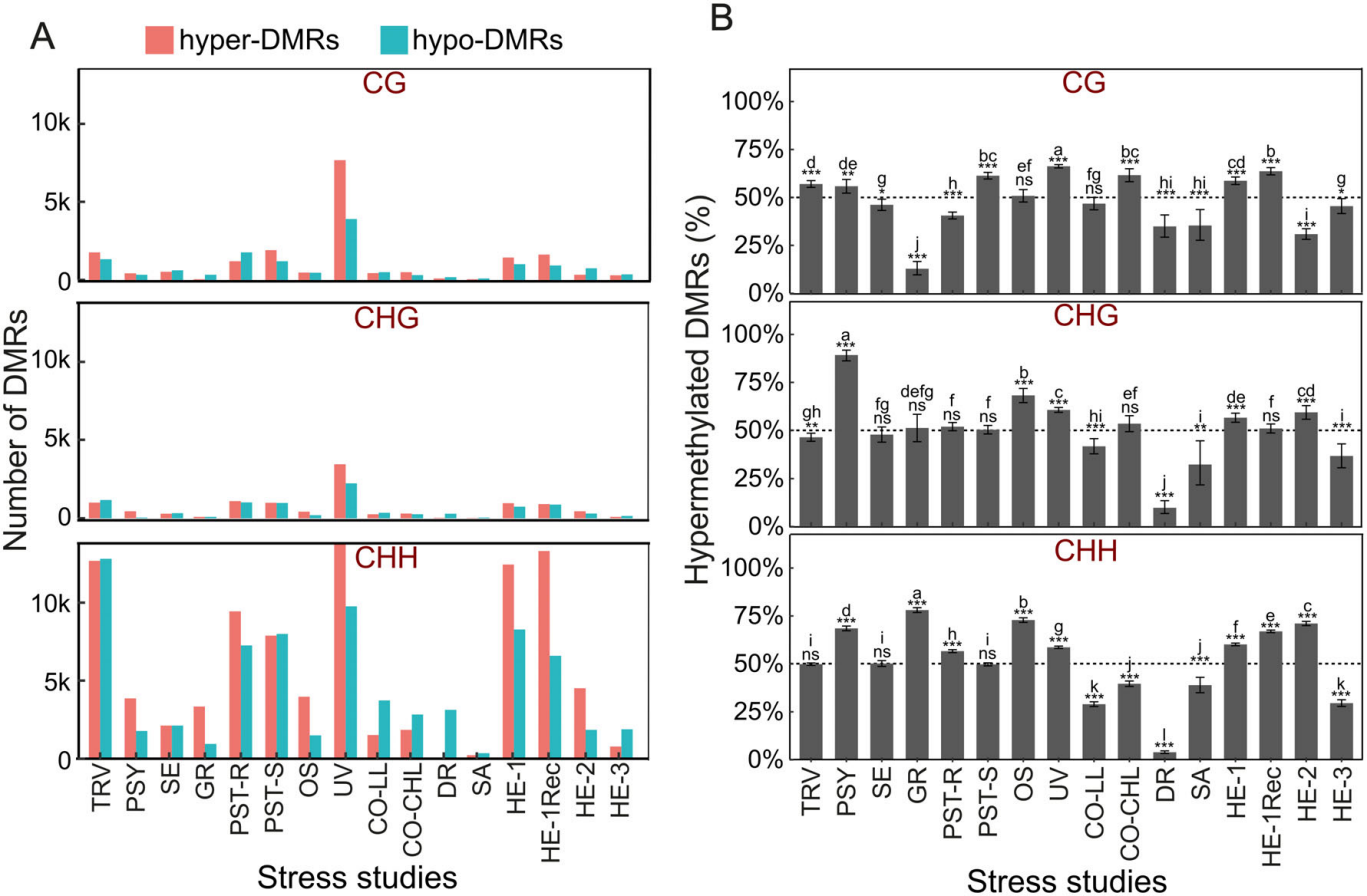
Distribution and relative proportion of hyper‐ and hypomethylated DMRs across Arabidopsis stress methylome datasets. (A) Number of hypermethylated and hypomethylated DMRs identified in each study in the *CG, CHG and CHH* sequence contexts. Bars show absolute DMR counts for each study, separated into hypermethylated and hypomethylated categories. (B) Proportion of *hypermethylated DMRs (%)* relative to the total number of directional DMRs (*hyper* + *hypo*) in each study for the *CG, CHG and CHH* contexts. Bars represent the percentage of hypermethylated DMRs, and error bars indicate 95% binomial confidence *intervals*. Statistical significance was evaluated by testing the observed proportion of hypermethylated DMRs against an expected 50:50 hyper/hypo distribution using exact binomial tests, followed by Benjamini–Hochberg correction for multiple testing.

In the CHG context, PSY showed the strongest hypermethylation bias, with nearly 90% of DMRs classified as hypermethylated. Additional hypermethylation biases were observed in HE‐1, HE‐2, OS and UV, whereas DR showed a strong hypomethylation bias. Several datasets, including GR, HE‐1 Recovery, PST‐R, PST‐S, SA and SE, did not deviate significantly from the 50:50 expectation. In the CHH context, GR, HE‐1, HE‐1 Recovery, HE‐2, OS, PST‐R, PSY and UV were significantly enriched for hypermethylated DMRs, whereas CO‐CHL, CO‐LL, DR, HE‐3 and SA showed significant hypomethylation bias. PST‐S, SE and TRV were close to a balanced hyper‐ and hypo‐ratio. Together, these results indicate that stress‐associated DMR directionality is not uniform across studies, but instead depends on both the stress condition and methylation context. Pairwise *post hoc* comparisons further confirmed that several studies differed significantly in the proportion of hypermethylated DMRs within each context (Fig. 2A,B; Fig. S2).

To further understand the distribution of DMRs across the *A. thaliana* genome, DMR density was analysed in 500‐kb sliding windows across all five chromosomes (Fig. 3). We observed that DMR density across the genome was broadly consistent across studies, with CHG and CHH‐DMRs clustering mainly in pericentromeric regions of chromosomes, while CG‐DMRs were more evenly distributed in chromosome arms (Fig. S3). However, some differences were discernible. For instance, when we compared normalized DMR densities in pericentromeric regions, both CHG and CHH contexts had significantly higher DMR densities than CG contexts on chromosomes 1, 2, 3 and 5 (paired Wilcoxon *P* < 0.05), whereas on chromosome 4, CHG remained above CG (Fig. 3; Figs. S4 and S5). Finally, splitting DMRs by direction (hyper‐ vs. hypomethylated) recapitulated the similar genomic distribution patterns (Fig. S6A,B).

**Fig. 3:**
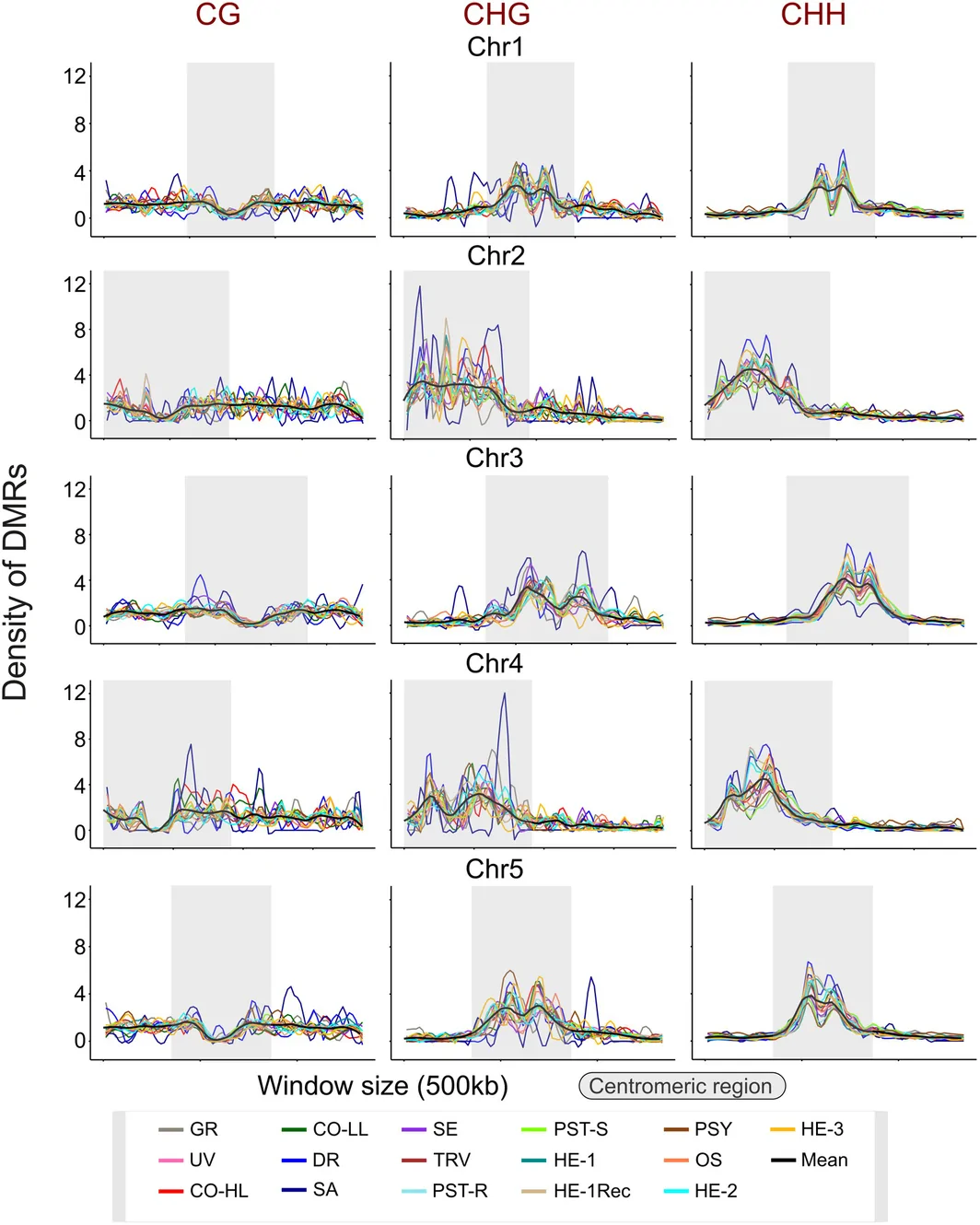
Density of DMRs per genomic window across studies. Densities are shown for three methylation contexts: CG, CHG and CHH. The grey segment highlights the centromeric region, where DMR density tends to peak in most contexts. Each line represents a different study, as indicated by the legend.

### Annotation‐centred DMR analysis

The characteristic density distribution of DMRs suggests that stress‐induced differential methylation shows a strong preference for specific genomic features. To examine this in more detail, we annotated all DMRs for their association with genes, TEs, promoters, intergenic regions and non‐coding RNAs (ncRNAs). As expected, most CG‐DMRs were associated with genes, whereas CHG‐DMRs and CHH‐DMRs were predominantly linked to TEs, followed by intergenic regions (Fig. 4A–C). To test this statistically, each DMR was assigned to a mutually exclusive primary annotation class, and chi‐square tests were performed using DMR counts. The distribution of DMRs across genomic features differed significantly among methylation contexts (χ^2^ = 54,773.74, df = 12, *P* < 0.001, Cramer's V = 0.355), confirming that CG‐, CHG‐ and CHH‐DMRs occupy distinct genomic compartments. Consistent with the visual pattern in Fig. 4, CG‐DMRs were predominantly associated with genes, with an average of 70.5% of CG‐DMRs assigned to gene regions across studies. In contrast, CHG‐ and CHH‐DMRs were more frequently associated with TEs, representing 33.9% and 42.2% of DMRs, respectively, followed by intergenic regions.

**Fig. 4:**
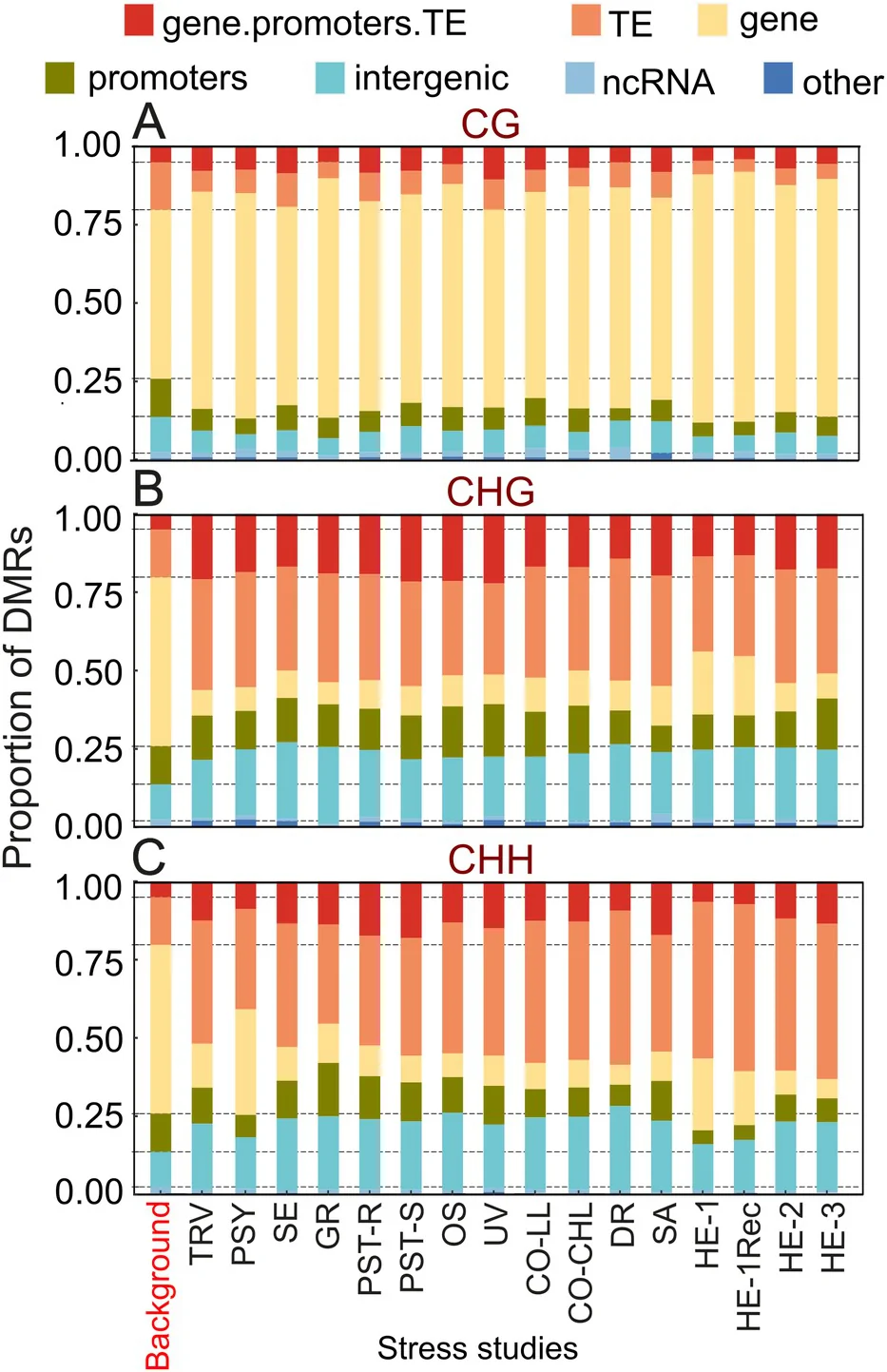
Proportion of DMRs annotated to different genomic features (*e.g*., gene body, promoter, intergenic regions, etc.) for each study. The three methylation contexts (A) CG, (B) CHG and (C) CHH are analysed separately. The ‘Background’ bar represents the genomic distribution of features across the entire genome for comparison.

We next tested whether annotation profiles differed among stress studies within each methylation context. Study‐by‐annotation chi‐square tests were significant for CG, CHG and CHH contexts (CG: χ^2^ = 715.22, *P* = 0.0001, Cramer's V = 0.060; CHG: χ^2^ = 587.92, *P* = 0.0001, Cramer's V = 0.070; CHH: χ^2^ = 11,519.59, *P* = 0.0001, Cramer's V = 0.108) (File S1). However, the effect sizes were small, indicating that although statistically detectable differences exist among studies, the dominant context‐specific annotation pattern is broadly conserved. Pairwise comparisons further showed that CHH‐DMR annotation profiles were the most variable across studies, with PSY showing the largest differences compared with other datasets. Together, these analyses support the conclusion that CG‐DMRs are mainly gene‐associated, whereas non‐CG‐DMRs are preferentially associated with TEs and intergenic regions, with limited but detectable study‐specific deviations.

A similar overall pattern was observed when hypermethylated and hypomethylated DMRs were analysed separately. In both directions, genomic‐feature distributions differed significantly among methylation contexts (hyper‐DMRs: χ^2^ = 27,632.61, df = 12, *P* < 0.001, Cramer's V = 0.390; hypo‐DMRs: χ^2^ = 19,737.91, df = 12, *P* < 0.001, Cramer's V = 0.356), confirming that the context‐dependent distribution of DMRs was retained after separating methylation direction. Hyper‐ and hypo‐DMRs also differed significantly within each context, but the effect sizes were small (Cramer's V = 0.058–0.081), indicating that direction‐specific differences were limited compared with the stronger effect of methylation context. Pairwise study comparisons showed that annotation profiles varied most strongly in the CHH context, especially among hypo‐DMRs, whereas CHG hypo‐DMR profiles were comparatively conserved. Together, these results show that CG‐DMRs remain predominantly gene‐associated, while CHG‐ and CHH‐DMRs are more frequently associated with TEs and intergenic regions, with additional stress‐specific deviations (Fig. S7A,B and File S1).

### Gene‐based DMR analysis

To identify genes responsive to multiple stress conditions, we analysed CG‐DMRs in genes across studies. In total, we found 10,639 non‐redundant genes harbouring at least one CG‐DMR. Among these, 59.4% (6,320 genes) appeared in at least two studies, and 33.3% (3,542 genes) were shared by three studies (Fig. 5A; File S2). The number of shared genes decreased with increasing study overlap: 15 genes were present across nine studies, and a single gene, *AT1G67500* (REV3), was present across ten studies.

**Fig. 5:**
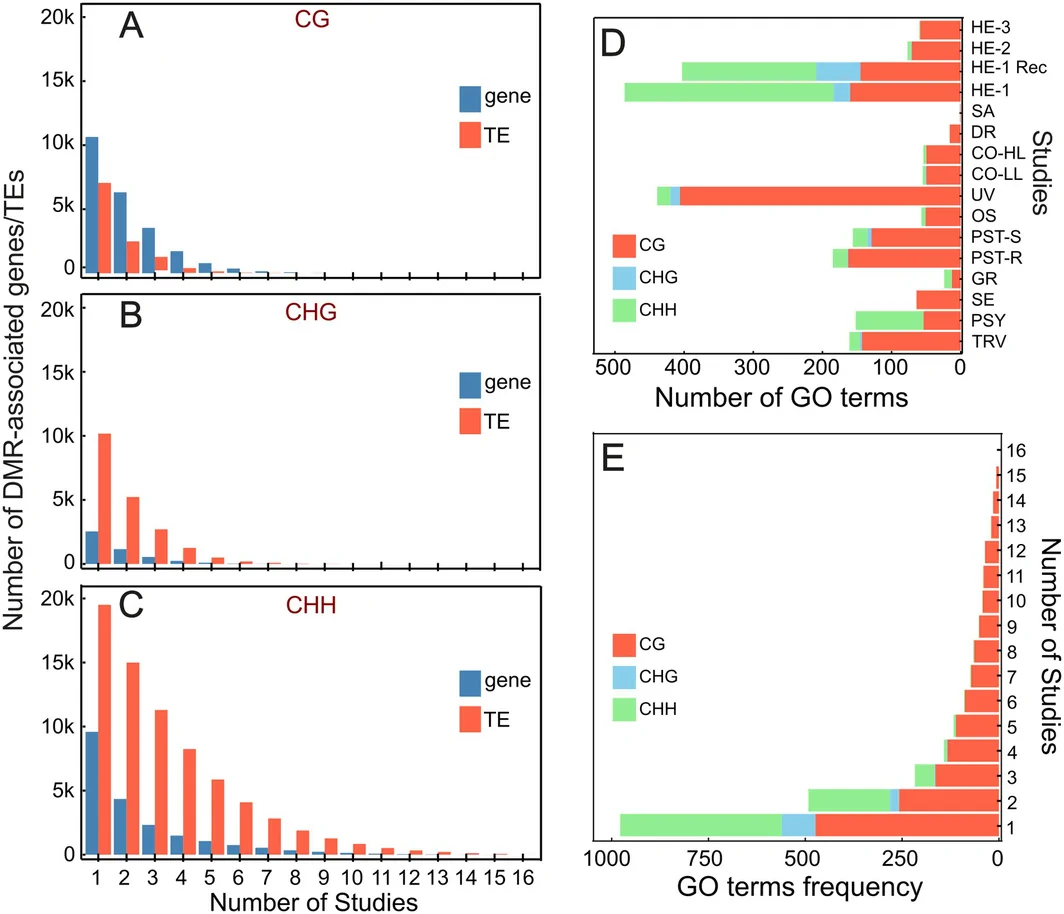
Number of DMR‐associated genes and TEs shared across studies. Bar plots show the number of DMR‐associated genes (blue) and TEs (red) identified in pairwise comparisons of studies. Each panel corresponds to one of the three methylation contexts: (A) CG, (B) CHG and (C) CHH. The *x*‐axis represents the number of studies in which DMR‐associated features are shared, while the *y*‐axis indicates the count of shared genes or TEs. (D) Number of functional categories (biological processes, BP; molecular function, MF; cellular component, CC) enriched for DMRs in each study. Bars are grouped by study and colour‐coded to represent the three methylation contexts: CG (orange), CHG (blue) and CHH (green). (E) Frequency distribution of enriched Gene Ontology (GO) terms shared across studies for DMR‐associated genes in the CG (orange), CHG (blue) and CHH (green) methylation contexts. The *y*‐axis represents the number of GO terms, and the *x*‐axis indicates the number of studies sharing the enriched terms across the three GO categories: BP, MF and CC.

In comparison, CHG‐DMR yielded 2,540 unique annotated genes. Among these, 45.7% (1,159 genes) were shared across at least two studies, and 21.6% (548 genes) were common among three studies (Fig. 5B; File S2). Genes such as *AT3G45140* (chloroplast lipoxygenase LOX2) and *AT1G33950* (Immune‐associated nucleotide (IAN)‐binding protein 7) were reported in 10 and 8 studies, respectively (TAIR, 2024).

In the CHH context, 9,589 annotated genes overlapped with DMRs. Among these, 45.4% (4,350 genes) were shared among at least two studies, and 24.3% (2,326 genes) were common among three studies (Fig. 5C; File S2). Interestingly, four genes were shared across 15 studies: *AT2G02635* (unknown function), *AT2G07240* (cysteine‐type peptidase), *AT2G10625* (Ulp1 protease family), and *AT2G11851* (unknown function), and one gene (*AT2G07240*‐ cysteine‐type peptidase) was shared across all 16 studies.

To assess whether DMR‐associated genes identified in each sequence context are part of a conserved regulatory response, we performed a bootstrap analysis using all annotated Arabidopsis genes as a background (Berardini *et al*. 2015; Araport11). We found that the overlapping set of DMR‐associated genes was non‐random across studies (Bootstrap *P* < 0.0001), indicating that DNA methylation responses to diverse stressors converge on a set of shared methylation‐responsive genes (Fig. S9). A similar pattern was observed when analysing hyper‐ and hypomethylated DMRs separately (Figs. S8 and S10 and Files S10 and S11).

### Functional enrichment of DMR‐associated genes

To investigate biological processes potentially regulated by differential methylation, we used g:Profiler to perform GO‐term enrichment analysis on the differentially methylated genes from each stress study. Various enriched GO terms (functional categories) were identified for all the studies in the CG context. However, in some studies in the CHG (GR, SE, PSY, OS, HE‐3, CO‐HL, DR and SA) and CHH (SE, DR and SA) contexts, no significantly enriched GO terms were detected. In general, more enriched GO terms were found in the CG context due to the greater number of genes identified in this context (Fig. 5D). Specifically, the highest number of enriched GO terms was observed in the HE‐1 and HE‐1 Recovery studies.

To analyse overlapping stress responses, enriched GO terms were compared across studies. In the CG context, 474 unique/non‐redundant enriched GO terms were identified after pooling all study‐specific enrichment results and removing duplicate GO terms. Of these, 258 GO terms were detected in at least two studies, and 164 were detected in at least three studies. Seven GO terms were highly recurrent, being shared by 15 study‐specific CG enrichment lists, and were mainly related to binding processes, including anion binding and ATP binding (Fig. 5D–E; File S3). In the CHG context, 87 unique/non‐redundant enriched GO terms were identified across all studies, with only a limited number shared across multiple studies; the most recurrent terms were associated with cell‐wall structure and extracellular regions (Fig. 5D–E; File S4). In the CHH context, 417 unique/non‐redundant enriched GO terms were identified, with recurrent terms mainly associated with cell‐wall structure and hydrolase activity (Fig. 5D–E; File S5).

Stratification of DMR‐associated genes into gbM and non‐gbM classes revealed distinct enrichment patterns. Non‐gbM genes showed broader enrichment across CHG and CHH contexts, whereas gbM‐associated enrichments were more limited (Fig. S12A–C). A Fisher's exact test further supported this differential pattern of enrichment (one‐sided Fisher's exact test; *P* < 0.05). Non‐gbM genes in the CG context were particularly enriched for GO terms (top 12) related to cellular organization and metabolic processes. In contrast, enriched GO terms among gbM genes were primarily associated with basic cellular processes such as enzymatic activities (*e.g*., kinase activity or phosphorylation). For the CHH context, significant non‐GbM GO terms were predominantly associated with cell‐wall structure, extracellular regions and hydrolase enzyme activity. These results suggest that differential methylation events, especially in non‐gbM genes, target specific stress‐responsive pathways (Fig. S12D–F).

We used a network‐based analysis to further visualize the relationships between stress conditions and enriched GO terms (Fig. 6). In the CG context, UV stress showed the highest number of enriched GO terms, especially those related to binding (*e.g*., ATP, adenyl nucleotide, anion, carbohydrate) and enzymatic activities (ATP hydrolysis, catalytic activity, transferase activity). TRV, PST‐R, PST‐S and HE‐1 also shared many of these terms, whereas SA and DR exhibited limited GO terms, with SA enriching only ATP‐dependent activity and DR highlighting GABA biosynthesis and cell‐surface receptor signalling (Fig. 6A; File S6).

**Fig. 6:**
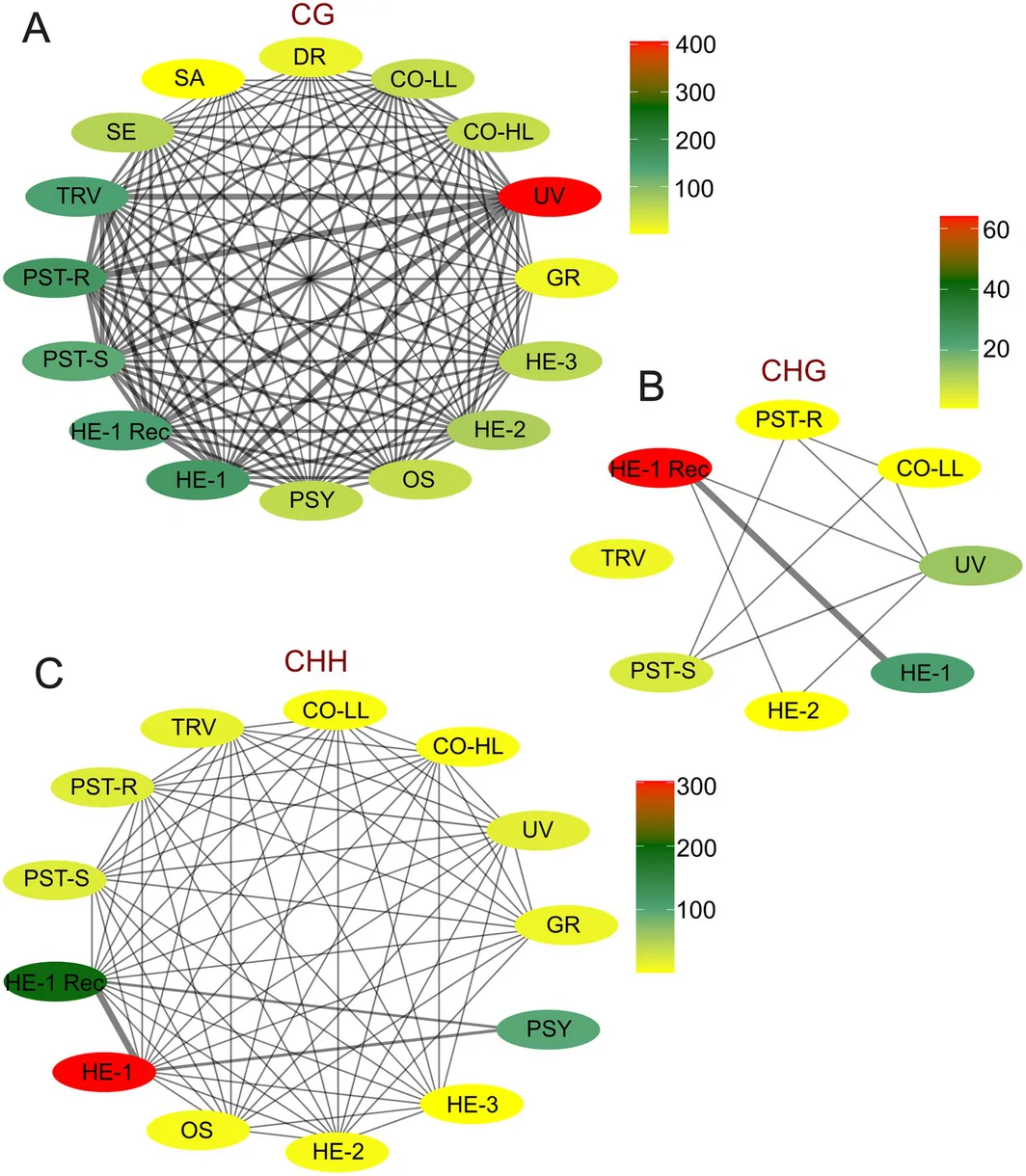
Network graphs illustrating the relationships between studies based on enriched GO categories. The node colours correspond to the number of GO categories enriched in each study (green: fewer categories; red: more categories). The edge widths indicate the number of shared GO terms between studies, with thicker edges representing greater overlap. (A) CG context (B) CHG context (C) CHH context.

In the CHG context, HE‐1 and HE‐1 Recovery had the most enriched terms—anchoring and cell‐wall junctions and structural molecule activities—reflecting cell‐wall modifications during heat stress. Wall constituents, whereas TRV showed no overlap (Fig. 6B; File S6). In CHH, HE‐1, HE‐1 Recovery and PSY were prominent for cell‐wall structure, cellular organization and enzymatic activities; PSY, bacterial and external stimulus responses, and UV, DNA polymerase activity (Fig. 6C; File S6). Overall, these GO‐term enrichments reveal both shared and stress‐specific methylation‐driven processes—particularly those governing binding functions, cell‐wall integrity and enzymatic pathways—highlighting conserved molecular mechanisms that underpin plant adaptation to diverse stress conditions.

### TE‐based DMR analysis

To identify differentially methylated TEs shared across stress studies, we collated 7,051 CG‐DMR‐associated TEs and found that 2,489 (35.3%) were shared by at least two studies, dropping to 1,045 (14.8%) in three studies (Fig. 5A; File S1). Notably, four TEs (*AT1TE48745/LTR/Gypsy*, *AT1TE50315/LTR/Gypsy*, *AT1TE50375/LTR/Gypsy*, *AT1TE50810/LTR/Gypsy*) recurred in seven studies, and *AT1TE50315* appeared in eight studies.

In the CHG context, 10,179 CHG‐DMR‐associated TEs were detected, of which 5,229 (51.4%) were shared by ≥2 studies and 2,702 (26.5%) by ≥3 (Fig. 5B). Three CHG‐TEs (AT2TE22065/LTR/Gypsy, AT3TE51685/LTR/Gypsy, AT5TE35915/RC/Helitron) were present in ten studies, and two (AT2TE22065, AT3TE51685) in eleven (Fig. 5B; File S2). The CHH context showed the highest level of sharing: of 19,504 CHH‐DMR‐associated TEs, 14,997 (76.9%) occurred in ≥2 studies and 11,300 (57.9%) in ≥3 (Fig. 5C). Eight CHH‐TEs (*e.g*., AT2TE00015/LTR/Gypsy, AT2TE10385/RC/Helitron, AT2TE11515/DNA/MuDR, AT2TE16220/RC/Helitron, AT2TE17095/RC/Helitron, AT4TE17970/RC/Helitron, *AT5TE45920*/RC/Helitron, *AT5TE47895*/RC/Helitron) were common to all 16 studies (Fig. 5C; File S2).

To assess whether DMR‐associated TEs identified in each sequence context are part of a conserved regulatory response, we performed a bootstrap analysis using the full complement of ~32,000 annotated Arabidopsis TEs (Fig. S9) (Quesneville 2020). We found that the overlapping set of DMR‐associated TEs was non‐random across studies (Bootstrap *P*‐value < 0.0001), indicating that DNA methylation responses to diverse stressors converge on a set of shared methylation‐responsive TEs (Fig. S9). A similar pattern was observed when analysing hyper‐ and hypomethylated DMRs separately (Figs. S8 and S11, and Files S10 and S11).

### Gene‐TE proximal DMR analysis (& GO analysis of genes in close proximity to TEs)

To determine the relationship between genes and TEs in close proximity, we analysed the density of TEs relative to their distance from nearby genes. The results (Fig. S13A) indicated that 25% of all TEs were located within 1.2 kb of the nearest gene, both upstream and downstream, while 50% were within 1.7 kb. We further examined TE superfamilies for different distance subsets (25%, 50%, 75% and 100%) and calculated the proportion of sequence space occupied by each superfamily (Fig. S13B). Among TEs located near genes, RC/Helitron was the predominant superfamily, accounting for 46% of the total TEs in the 25% subset and increasing to 61% in the 75% subset. However, when considering all TEs (100% subset), the proportion of RC/Helitron decreased to 32%. Overall, the majority of differentially methylated TEs near genes belonged to the RC/Helitron, DNA/MuDR, LTR/Copia and LTR/Gypsy superfamilies.

To investigate the enrichment of differentially methylated TEs near genes, we restricted our analysis to all TEs located within 1.2 kb (25% subset) of an annotated gene. For each TE superfamily and each stress study, we then calculated the proportion of these proximal TEs overlapping DMRs in CG, CHG and CHH sequence contexts. To assess statistical significance, we performed 10,000 permutations of the DMR‐overlap labels across that same fixed set of proximal TEs, yielding a null distribution of enrichment for each family. In the CG context, the highest enrichment (Δ% true overlap vs. the permuted overlap) was seen for LTR/Copia in TRV, PST‐R, UV and CO‐CHL; RC/Helitron in PSY, PST‐R and UV; DNA/MuDR in PST‐R and UV; DNA/Pogo in HE‐1 recovery; and DNA/HAT in HE‐3 (*P* < 0.01 for each). This indicates that, among TEs within 1.2 kb of genes, these superfamilies are significantly overrepresented among those overlapping CG‐DMRs (Figs. S14 and S15).

In the CHG context, DNA/MuDR, LTR/Copia and RC/Helitron superfamilies were each significantly overrepresented among gene‐proximal CHG‐DMRs (all *P* < 0.05). In the CHH context, we observed a distinct pattern: DNA/HAT, DNA/En‐Spm, DNA/MuDR, LTR/Gypsy and RC/Helitron were all significantly enriched for CHH‐DMRs (*P* < 0.05).

Strikingly, across all three sequence contexts, the families DNA/HAT, DNA/MuDR and RC/Helitron comprised a conserved core of stress‐responsive elements. By contrast, other superfamilies, such as DNA/Harbinger, DNA/T1 and LINE/L1, showed significant enrichment only under specific treatments (*e.g*., DNA/Harbinger in UV for CHH; LINE/L1 in HE‐2 for CHH), underscoring both a universal TE–methylation signature and treatment‐specific dynamics (Fig. 7).

**Fig. 7:**
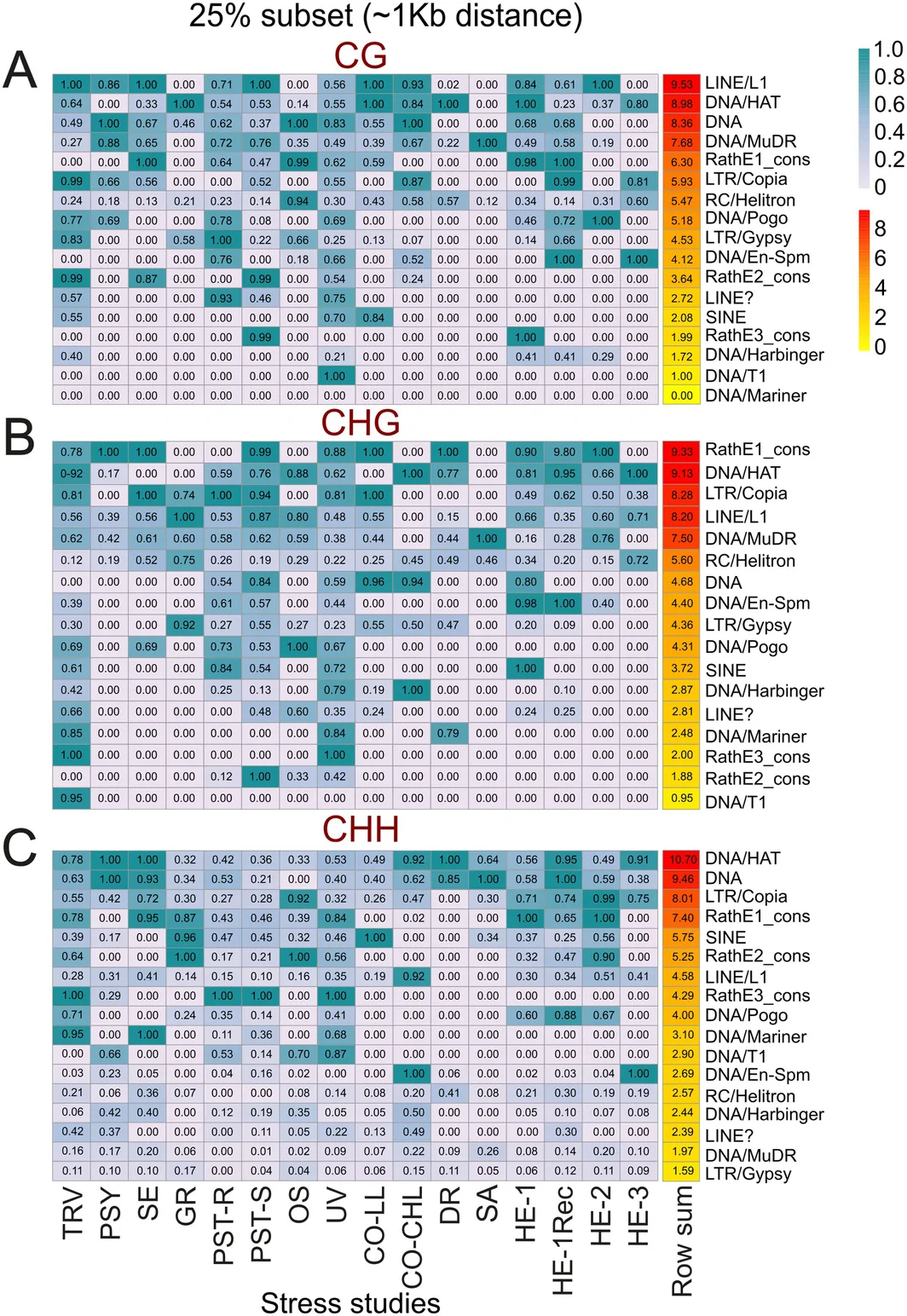
Heatmap showing TE superfamilies located within 25% (approximately 1.2 kb) of DMR‐associated genes, sorted in descending order of their enrichment for (A) CG, (B) CHG and (C) CHH contexts. The colour gradient reflects the level of enrichment, with red indicating higher enrichment and blue indicating lower enrichment.

We next asked whether genes bearing nearby TE‐DMRs might have documented stress‐related functions. Across all three sequence contexts, GO‐enrichment of proximal genes highlighted core processes‐binding and enzymatic activities; transport; reproduction/development; defence; cell‐wall biogenesis; and notably, chromatin remodelling in CG and ncRNA regulation in CHH (Fig. 8A–C; Files S7–S9 and S12).

**Fig. 8:**
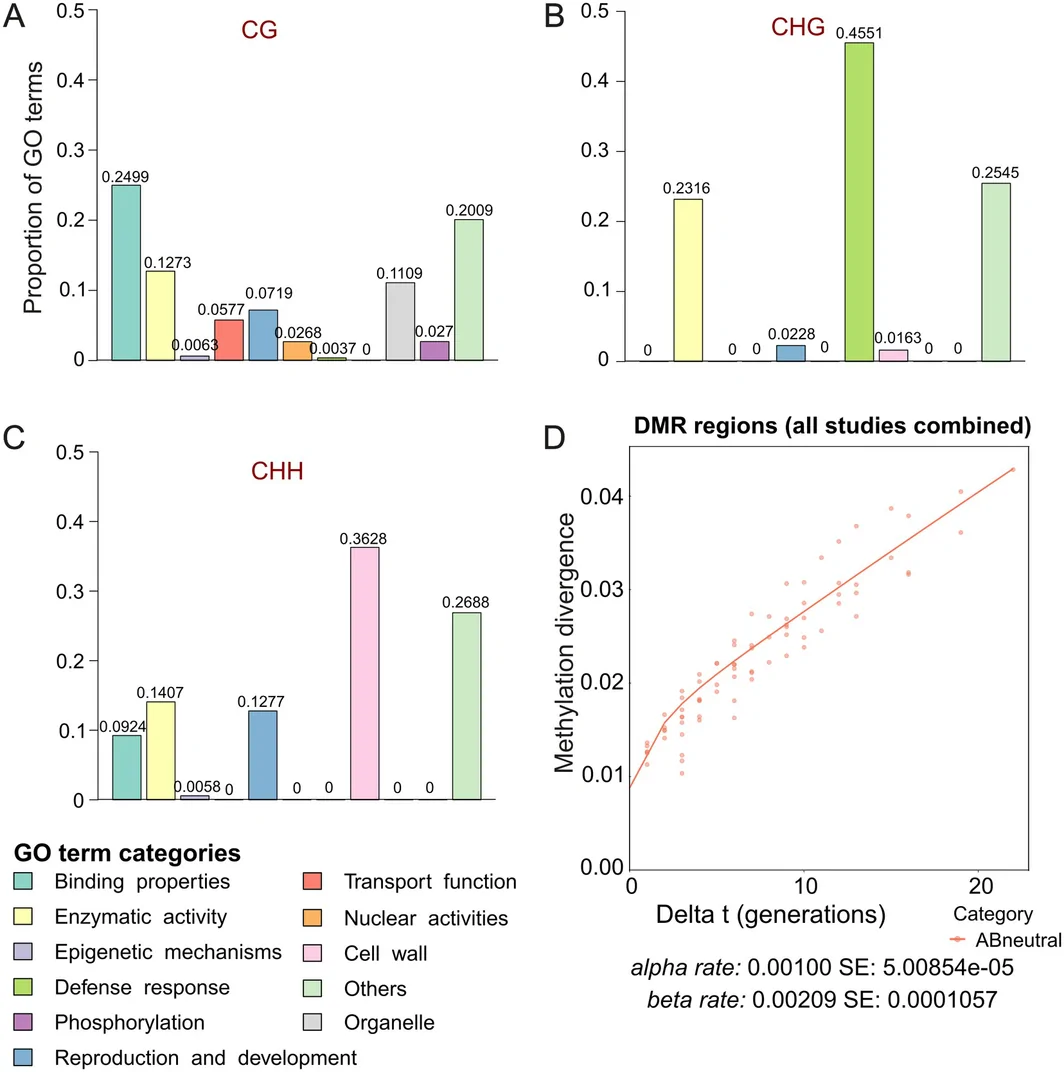
Histograms showing the results of functional enrichment analysis for genes located in 1Kbp close proximity to differentially methylated TEs. The *x*‐axis represents functional categories or GO terms, and the *y*‐axis represents the number of genes associated with each category. (A) Genes near TEs in the *CG* methylation context. (B) Genes near TEs in the *CHG* methylation context. (C) Genes near TEs in the *CHH* methylation context. (D) *AlphaBeta analysis* illustrates the relationship between 5‐methylcytosine (5mC) divergence and delta time, describing the accumulation of spontaneous epimutations over successive generations. The *x*‐axis represents delta time, while the *y*‐axis indicates methylation divergence. Data points reflect observed values, and the trendline highlights the pattern of spontaneous epimutation accumulation.

### Potential for transgenerational inheritance of stress‐induced CG methylation

While stress‐induced DNA methylation changes are often considered transient—typically reprogrammed during reproduction – emerging evidence suggests that some of these changes, particularly in the CG context, may persist across generations (Johannes & Schmitz 2019). This raises the possibility that stress could contribute to long‐term, heritable epigenetic variation. As our DMR analysis showed, most stress‐responsive methylation changes occur in TEs, which are targeted by the RNA‐directed DNA methylation (RdDM) pathway (Law *et al*. 2010, 2013; Matzke & Mosher 2014). This pathway reinforces methylation during gametogenesis, thereby resetting most environmentally induced changes and preventing their transgenerational inheritance. However, not all genomic regions are under strong RdDM control. Recent studies have shown that CG sites outside of RdDM targets can undergo spontaneous epimutation; that is, heritable stochastic gains or losses of methylation that arise during imperfect methylation maintenance in mitosis or meiosis (Becker *et al*. 2011; Schmitz *et al*. 2011; Schmitz & Ecker 2012; Van Der Graaf *et al*. 2015; Hofmeister *et al*. 2020; Denkena *et al*. 2021; Hazarika *et al*. 2022; Yao *et al*. 2023). These CG epimutations, unlike their CHG and CHH counterparts, can accumulate over generations at rates far exceeding those of genetic mutations, offering a substrate for long‐term epigenetic divergence. We hypothesized that CG‐DMRs identified in stress response studies may overlap with genomic regions prone to spontaneous epimutations, providing a mechanistic link between stress‐induced methylation and stable, heritable changes in gene regulation. Although direct transgenerational data are lacking for the stress datasets we analysed, we assessed the potential stability of stress‐responsive CG‐DMRs by examining their behaviour in Arabidopsis wild‐type mutation accumulation (MA) lines (Yao *et al*. 2023). These lines, derived from single founders and propagated by selfing for 17 generations, provide a robust system for studying epimutation accumulation and estimating epimutation rates. We focused our analysis on CG‐DMRs identified in the comparative stress dataset and observed that CG sites within these regions accumulate epimutations over successive generations (Fig. 8D; File S13). This finding indicates that methylation changes—whether arising spontaneously or triggered by environmental stress—can persist over time, serving as a potential interface between external stress cues and long‐term epigenetic inheritance. Such stability suggests a mechanism by which environmental conditions may leave a heritable molecular imprint, potentially influencing adaptive trajectories across generations.

### Metadata‐stratified subgroup analysis reveals recurrent annotation‐level methylation signatures

To address experimental heterogeneity among the included methylome studies, we performed a metadata‐stratified analysis, grouping studies by stress type or tissue similarity into heat stress, phosphate starvation, biotic stress and leaf/shoot‐derived subgroups. Within each subgroup, DMR‐associated genes and TEs were separately compared across CG, CHG and CHH contexts. Most differentially methylated genes and TEs detected were unique to each study, indicating substantial study‐specificity. However, a subset of features (genes/TEs) was recurrent within defined subgroups, particularly in the CHH context and in the leaf subgroup (Figs. S16–S18).

We next identify recurrent functional categories within the same defined subgroups. Subgroup‐consensus GO terms in the CG context were mainly associated with broad binding‐related functions, including ATP binding, nucleotide binding, ribonucleotide binding and carbohydrate derivative binding. In the CHH context, recurrent GO terms included structural constituents of the cell wall, particularly in the leaf subgroup (Fig. S19D).

Finally, we focused specifically on gene‐proximal TEs, as these elements may be more likely to influence the regulatory environment of nearby genes. Subgroup‐consensus gene‐proximal DMR‐associated TEs showed a strong context‐dependent pattern. In the CG context, recurrence was limited and mainly represented by DNA/Pogo and DNA/HAT. In the CHG context, DNA/HAT, RathE1_cons and DNA/Pogo remained the most recurrent superfamily, with additional contributions from RC/Helitron. The strongest recurrence was observed in the CHH context, where DNA/HAT, LTR/Gypsy, DNA/MuDR and RathE3_cons elements were repeatedly detected across defined subgroups (Fig. S19A–C).

## DISCUSSION

Our reanalysis of *Arabidopsis thaliana* methylomes under diverse biotic and abiotic stresses provides a comparative view of how DNA methylation responds to environmental perturbation across independent studies. Although global CG, CHG and CHH methylation levels remained largely stable, localized DMRs revealed clear context‐ and stress‐dependent variation. This supports the view that genome‐wide methylation patterns are generally buffered by robust homeostatic mechanisms, whereas specific genomic regions remain responsive to environmental change (Kawakatsu *et al*. 2016; Bewick & Schmitz 2017). The observed divergence in DMR directionality across stress contexts, including CHG hypermethylation under *Pseudomonas syringae* infection and hypomethylation under drought and gamma irradiation, is consistent with previous reports that different stresses can activate distinct methylation pathways (Dowen *et al*. 2012; Ganguly *et al*. 2017).

The genomic distribution of DMRs further emphasized the context‐specific nature of stress‐associated methylation. CG‐DMRs were more frequently associated with genes and chromosome arms, whereas CHG‐ and CHH‐DMRs were enriched in TE‐rich and pericentromeric regions. This pattern is consistent with the established role of non‐CG methylation in heterochromatin and TE silencing and with previous studies linking heterochromatic methylation to genome stability under stress (Zemach *et al*. 2013; Stroud *et al*. 2014). Although global methylation levels remained stable, stress‐responsive DMRs were not randomly distributed across the genome but were concentrated in genomic compartments with distinct regulatory functions.

Several recurrent DMR‐associated genes identified in our analysis have previously been linked to stress‐related processes, supporting the biological relevance of the shared methylation signatures. For example, AT1G67500 encodes REV3, the catalytic subunit of DNA polymerase ζ, which has been associated with translesion DNA synthesis and UV/gamma radiation tolerance (TAIR, 2024 and Sakamoto *et al*. 2003). Similarly, AT3G45140 encodes LOX2, a chloroplast lipoxygenase implicated in jasmonate‐mediated responses to wounding, UV and osmotic stress, while AT1G33950 encodes a member of the IAN/AIG1 family, which has been associated with immune‐related responses (Chauvin *et al*. 2013; Lv *et al*. 2019; Chen *et al*. 2021; Singh *et al*. 2022). The recurrence of these genes across independent stress datasets suggests that stress‐associated DMRs may repeatedly affect loci involved in DNA damage tolerance, jasmonate signalling, proteolysis and immune‐related pathways (Brocklehurst *et al*. 2018).

Functional enrichment analysis further indicated that DMR‐associated genes are not randomly distributed across biological processes. In particular, non‐gbM genes showed stronger enrichment for stress‐related functional categories than gbM genes. This is consistent with previous reports that non‐gbM genes are generally more responsive to environmental and regulatory perturbation, whereas gbM genes are often associated with more constitutive or housekeeping functions (Horvath *et al*. 2019; Muyle *et al*. 2022; Zhang *et al*. 2024). The enrichment of cell‐wall‐related terms, especially in heat‐stress datasets, suggests cell‐wall remodelling, extracellular structure and hydrolase activity as important components of abiotic stress acclimation (Le Gall *et al*. 2015). Together, these patterns indicate that stress‐induced methylation changes preferentially affect gene classes and pathways with regulatory plasticity.

TEs emerged as a major component of the stress‐responsive methylome. This is expected because TE activity is tightly controlled by DNA methylation, particularly in CHG and CHH contexts, to maintain genome stability (Lisch 2013; Secco *et al*. 2015; Chuong *et al*. 2017). At the same time, TEs can contribute to stress‐responsive gene regulation by carrying cis‐regulatory motifs or influencing nearby gene expression (Dubin *et al*. 2018; Deneweth *et al*. 2022). The strong recurrence of differentially methylated TEs across datasets therefore supports the idea that TE methylation is a common target of stress‐associated epigenetic regulation. The repeated detection of specific LTR/Gypsy elements across multiple studies is consistent with previous observations that some Arabidopsis TE insertions are repeatedly differentially methylated across accessions or environmental conditions (Stuart *et al*. 2016; Arora *et al*. 2022).

Our gene‐proximal‐TE analysis further suggests that stress‐associated TE methylation may have potential regulatory relevance. Several TE superfamilies enriched near genes, including RC/Helitron, DNA/MuDR, DNA/HAT and DNA/Pogo, have not been widely discussed as stress‐responsive methylation targets in Arabidopsis. This broadens the focus beyond well‐characterized heat‐responsive elements such as ONSEN LTR/Copia, which is transcriptionally activated under elevated temperature and can influence nearby gene expression (Ito *et al*. 2011; Pietzenuk *et al*. 2016). The identification of recurrent genes near TE‐DMRs, including *AT3G44570, WRKY42* and *AT4G16860/RPP6*, further supports a possible link between TE‐proximal methylation and stress or immune responses. *AT3G44570* encodes a retrotransposon ORF1‐related protein involved in L1 ribonucleoprotein formation, *WRKY42* belongs to a transcription factor family broadly involved in stress and defence regulation, and RPP6 is associated with pathogen resistance in Arabidopsis (Van Der Biezen *et al*. 2002; Martin 2006; Phukan *et al*. 2016). Although methylation changes alone do not demonstrate transcriptional regulation, these findings identify candidate loci where TE‐associated methylation may intersect with stress‐responsive gene regulation.

One notable example is *AT5TE35120* near the CNI1 locus. Previous work showed that methylation changes at this TE are associated with CNI1‐AS1 expression across generations exposed to salt stress (Wibowo *et al*. 2016, 2018). In our analysis, *AT5TE35120* was hypomethylated in the CG context in the high‐osmolarity dataset, supporting the idea that some stress‐responsive TE‐DMRs may be linked to nearby gene regulation. This example illustrates how reanalysis across studies can recover known stress‐associated methylation events while also identifying additional candidate TE families and loci for future functional validation.

The metadata‐stratified subgroup analysis refined the conclusions from the full cross‐study comparison. In the complete dataset, methylation changes showed strong context‐dependent genomic distributions: CG‐DMRs were mainly associated with genes, whereas CHG/CHH‐DMRs were more frequently associated with TEs and intergenic regions. The subgroup analysis showed that these broad patterns were retained when studies were grouped by more comparable stress or tissue metadata. Although many DMR‐associated genes and TEs remained study‐specific, recurrent features were detected within subgroups, particularly in the CHH context and among gene‐proximal TEs. DNA/HAT, DNA/Pogo, DNA/MuDR and LTR/Gypsy elements were especially recurrent among subgroup‐consensus gene‐proximal DMR‐associated TEs, suggesting that non‐CG methylation changes repeatedly affect similar TE annotations across comparable datasets. Functional recurrence was also consistent with the full analysis, with CG subgroup‐consensus GO terms dominated by binding‐related functions and CHH terms including cell‐wall‐associated processes. Therefore, the subgroup analysis supports the main conclusion while adding nuance: stress‐associated methylation marks are not highly conserved at every individual locus, but they show reproducible patterns at the level of genomic annotations, TE superfamilies and functional categories.

Finally, our epimutation analysis suggests that a subset of stress‐associated CG‐DMRs overlaps with genomic regions capable of accumulating stable methylation variation. Stress‐induced methylation changes are often considered transient, particularly when they occur in non‐CG contexts or TE‐rich regions targeted by RNA‐directed DNA methylation (Lämke & Bäurle 2017; Zhang *et al*. 2018). However, CG methylation can be maintained through mitotic and meiotic divisions, and mutation accumulation studies have shown that CG epimutations can persist over multiple generations (Van Der Graaf *et al*. 2015; Shahryary *et al*. 2020). Our observation that stress‐associated CG‐DMRs overlap with epimutation‐prone regions suggests that some environmentally responsive methylation changes occur at loci with intrinsic potential for long‐term epigenetic stability. Importantly, we did not directly demonstrate transgenerational inheritance in the stress datasets analysed here. Rather, our analysis identifies candidate regions where stress‐associated methylation and spontaneous stable epimutability intersect.

Collectively, this study shows that environmental stress does not lead to global disruptions of DNA methylation patterns in Arabidopsis, but instead induces localized, context‐dependent methylation changes at genes, TEs and gene‐proximal regulatory regions. These changes converge on stress‐relevant pathways, TE‐associated regulatory elements and a subset of CG methylation sites with potential long‐term stability. A limitation of this analysis is that the included WGBS datasets were heterogeneous in stress intensity, exposure duration, tissue type, developmental stage and experimental design. This heterogeneity reflects the limited number of publicly available Arabidopsis stress methylome datasets and prevents a fully matched meta‐analysis based on identical stress conditions. Therefore, our study should be interpreted as a standardized multi‐study reanalysis aimed at identifying broadly recurrent methylation features, rather than as a strictly controlled comparison of identical stress treatments. Future studies with harmonized experimental designs and matched methylome‐transcriptome data will be necessary to more precisely distinguish stress‐specific, tissue‐specific and developmentally dependent methylation responses. Additionally, future work should integrate matched transcriptome data, targeted methylome editing and explicit multi‐generational stress experiments to determine which of these methylation changes have direct regulatory functions and which can persist across generations.

## AUTHOR CONTRIBUTIONS

Experiments were designed by FJ, analyses were performed by RB, JJGF and RRH, research articles were searched by FW and RRH, data interpretation was performed by RB, CL, FJ and the paper was written by RB with input from CL, FJ and JPS. Epimutation analysis was performed by ZZ. The written paper was reviewed by all the co‐authors.

## FUNDING INFORMATION

This work (for RB and CL) was supported by the Initiative and Networking Fund of the Helmholtz Association under the call HIRS, the EpiCrossBorders: International Helmholtz‐Edinburgh Research School for Epigenetics.

## CONFLICT OF INTEREST

Not declared.

## Supporting information


**Fig. S1.** Schematic representation of dataset selection and filtering process for *Arabidopsis thaliana* WGBS experiments identified from the NCBI‐SRA database and included in the meta‐analysis.
**Fig. S2.** Proportions of hyper‐ and hypomethylated regions in stress‐related DMRs. Each bar represents the relative proportions of hyper‐ and hypomethylated regions for specific study for (A) CG, (B) CHG and (C) CHH contexts.
**Fig. S3.** Genome‐wide distribution of stress‐responsive DMRs across methylation contexts. (A) *CG*, (B) *CHG* and (C) *CHH*. Within each panel, the five horizontal tracks correspond to chromosomes 1–5 (top to bottom). Each point marks the genomic position (midpoint) of a DMR detected in a given stress study; colours indicate the stress/study as shown in the key. Grey shaded boxes highlight the annotated centromeric/pericentromeric regions for each chromosome.
**Fig. S4.** Context‐specific DMR density in pericentromeric regions versus genome‐wide. Boxplots summarize the distribution of *normalized DMR density* (fold‐enrichment) for *CG*, *CHG* and *CHH* contexts. The genome was tiled into *non‐overlapping 500‐kb windows* (Chr1–Chr5). For each window, DMR density was computed as the number of *significant DMRs* overlapping the window divided by the window size and then *normalized by the genome‐wide mean* for that context (yielding a fold change; 1 = genome average). (A) *Pericentromeric region*: windows within the pericentromere of each chromosome are shown; (B) *Whole genome*: all windows on the chromosome are shown. Boxes show medians and interquartile ranges across windows; whiskers extend to 1.5× IQR, with points indicating outliers. *Paired Wilcoxon signed‐rank tests* (across matched windows) compare contexts; formatted *P*‐values are displayed above comparisons.
**Fig. S5.** Pairwise differences in DMR density across stress studies (Kruskal–Wallis framework). Heatmaps show pairwise comparisons of DMR density between studies for each methylation context—*CG* (left column), *CHG* (middle) and *CHH* (right)—separately by chromosome: *Chr1* (D‐F), *Chr2* (G‐I), *Chr3* (J‐L), *Chr4* (M‐O), *Chr5* (P‐R) and *Genome‐wide (all chromosomes pooled)* (A–C). For each chromosome/context, DMR density was tested across studies with a Kruskal–Wallis rank‐sum test; when the overall test was significant, *post hoc* pairwise Wilcoxon with Benjamini–Hochberg correction. Matrix cells display the adjusted *P*‐values (numbers in cells), and colour encodes −log10 (adjusted *p*) (white = not significant/high *p*; dark red = stronger evidence of difference/low *p*). Diagonals are self‐comparisons. Study abbreviations follow the legend used in the main figures.
**Fig. S6.** Density of DMRs per genomic window across studies. Densities are shown for three methylation contexts: CG, CHG and CHH. The grey segment highlights the centromeric region, where DMR density tends to peak in most contexts. Each line represents a different study, as indicated by the legend. (A) Hypermethylated (B) Hypomethylated.
**Fig. S7.** Proportion of DMRs annotated to different genomic features (*e.g*., gene body, promoter, intergenic regions, etc.) for each study. The three methylation contexts (CG, CHG, CHH) are analysed separately. The ‘Background’ bar represents the genomic distribution of features across the entire genome for comparison. (A) Hypermethylated (B) Hypomethylated.
**Fig. S8.** Number of DMRs (hyper and hypo) ‐associated genes and TEs shared across studies. Bar plots show the number of DMR‐associated genes (blue) and TEs (red) identified in pairwise comparisons of studies. Each panel corresponds to one of the three methylation contexts: CG, CHG and CHH. The *x*‐axis represents the number of studies in which DMR‐associated features are shared, while the *y*‐axis indicates the count of shared genes or TEs. (A) Hypermethylated (B) Hypomethylated.
**Fig. S9.** Cross‐study sharing of DMR‐associated features assessed by bootstrap. (A) Genes and (B) TEs for CG, CHG and CHH. Blue bars show the observed number of unique features present in 16 studies (*x*‐axis). Orange bars summarize the expected counts under a bootstrap null that preserves each study's list size (10,000 resamples drawn from the background set); black error bars indicate the 95% bootstrap interval. Text above bars gives one‐sided enrichment *P*‐values computed as the fraction of bootstrap replicates with counts ≥ the observed (‘n.s.’, not significant).
**Fig. S10.** Cross‐study sharing of DMR‐associated features assessed by bootstrap. (A) Hypermethylated and (B) Hypomethylated genes for CG, CHG and CHH. Blue bars show the observed number of unique features present in 16 studies (*x*‐axis). Orange bars summarize the expected counts under a bootstrap null that preserves each study's list size (10,000 resamples drawn from the background set); black error bars indicate the 95% bootstrap interval. Text above bars gives one‐sided enrichment *P*‐values computed as the fraction of bootstrap replicates with counts ≥ the observed (‘n.s.’, not significant).
**Fig. S11.** Cross‐study sharing of DMR‐associated features assessed by bootstrap. (A) Hypermethylated and (B) Hypomethylated TEs for CG, CHG and CHH. Blue bars show the observed number of unique features present in 16 studies (*x*‐axis). Orange bars summarize the expected counts under a bootstrap null that preserves each study's list size (10,000 resamples drawn from the background set); black error bars indicate the 95% bootstrap interval. Text above bars gives one‐sided enrichment *P*‐values computed as the fraction of bootstrap replicates with counts ≥ the observed (‘n.s.’, not significant).
**Fig. S12.** gbM vs non‐gbM composition of DMR‐associated genes and their GO enrichments. (A–C) For each methylation context (CG, CHG, CHH; panel headers), horizontal bars show, per study (*y*‐axis), the number of DMR‐associated genes classified as gbM vs non‐gbM (colours as in the inset key). (D–F) Networks of enriched GO terms for the same contexts, shown separately for gbM (left, blue) and non‐gbM (right, green). The central node denotes the gene class; red nodes are GO terms. Edge thickness is proportional to the odds ratio from Fisher's exact test; thicker edges indicate stronger enrichment. Unless otherwise noted, only the top 12 terms by odds ratio are displayed for readability, and terms meeting FDR <0.05 (Benjamini–Hochberg) are considered significant. *Note: No term passed FDR in CHG context, the top 12 terms by odds ratio are shown for visualization only (no significance implied)*.
**Fig. S13.** (A) Density plot showing the distribution of TEs in proximity to genes. The *x*‐axis represents the relative distance of TEs from nearby genes, with 0% and 100% corresponding to the start and end of the gene regions, respectively. Red dashed lines indicate quartiles (25%, 50% and 75%).
**Fig. S14.** Permutation analysis of gene‐proximal‐TE superfamilies with context‐specific DMRs across stresses.
(A) CG, (B) CHG, (C) CHH. Columns are stress studies and rows are TAIR10 TE superfamilies. Each circle summarizes, for a given study and superfamily, the percentage of gene‐proximal TEs (elements within ±1.2 kb of gene TSSs) that overlap at least one DMR in that context; bubble area is proportional to this percentage. Colour encodes −log10 of the two‐sided permutation *P*‐value obtained by shuffling DMR labels within the proximal‐TE universe (10,000 permutations per study); black stars mark *P* < 0.05. The TE universe was derived from TAIR10 annotations and then restricted to gene‐proximal elements.
**Fig. S15.** Pairwise differences in enrichment of gene‐proximal‐TE superfamilies with DMRs across studies. (A) CG, (B) CHG, (C) CHH. Rows are TAIR10 TE superfamilies; columns are all pairwise study comparisons. For each superfamily and comparison, the tile shows % of gene‐proximal TEs (Δ%\Delta\%Δ%) in that superfamily that overlap (≥1 DMR in study a) − (**% in study b). Colour encodes the signed Δ%\Delta\%Δ%—diverging scale; red = a>b, blue = a<b; white ≈0). Stars mark two‐sided permutation significance (*P* < 0.05) obtained by shuffling study labels within the pooled proximal‐TE set 10,000 times and recomputing Δ%\Delta\%Δ%. Gene‐proximal TEs were defined as elements within ±1.2 kb of genes.
**Fig. S16.** Metadata‐stratified sharing of DMR‐associated genes and TEs across study subgroups. Bar plots show the number of DMR‐associated genes and DMR‐associated TEs shared across studies within each metadata‐defined subgroup for the *CG, CHG and CHH* contexts. Columns correspond to the four subgroups (Biotic, Heat, Leaf tissue and Phosphate), and rows correspond to methylation contexts. The *x*‐axis indicates the number of subgroup studies in which a given gene or TE was detected, and the *y*‐axis shows the number of DMR‐associated features. Blue bars represent genes, and orange/red bars represent TEs.
**Fig. S17.** Metadata‐stratified sharing of hyper‐DMR‐associated genes and TEs across study subgroups. Bar plots show the number of hyper‐DMR‐associated genes and hyper‐DMR‐associated TEs shared across studies within each metadata‐defined subgroup for the *CG, CHG and CHH* contexts. Columns correspond to the four subgroups (Biotic, Heat, Leaf tissue and Phosphate), and rows correspond to methylation contexts. The *x*‐axis indicates the number of subgroup studies in which a given gene or TE was detected, and the *y*‐axis shows the number of DMR‐associated features. Blue bars represent genes, and orange/red bars represent TEs.
**Fig. S18.** Metadata‐stratified sharing of hypo‐DMR‐associated genes and TEs across study subgroups. Bar plots show the number of hypo‐DMR‐associated genes and hypo‐DMR‐associated TEs shared across studies within each metadata‐defined subgroup for the *CG, CHG and CHH* contexts. Columns correspond to the four subgroups (Biotic, Heat, Leaf tissue and Phosphate), and rows correspond to methylation contexts. The *x*‐axis indicates the number of subgroup studies in which a given gene or TE was detected, and the *y*‐axis shows the number of DMR‐associated features. Blue bars represent genes, and orange/red bars represent TEs.
**Fig. S19.** Subgroup analysis of gene‐proximal‐TE superfamilies and GO terms.

## Data Availability

The data supporting the findings of this study are available upon request from the corresponding authors. The data are not publicly available due to privacy or ethical restrictions.
